# Dual Endothelin Receptor Blockade Abrogates Right Ventricular Remodeling and Biventricular Fibrosis in Isolated Elevated Right Ventricular Afterload

**DOI:** 10.1371/journal.pone.0146767

**Published:** 2016-01-14

**Authors:** Eva Amalie Nielsen, Mei Sun, Osami Honjo, Vibeke E. Hjortdal, Andrew N. Redington, Mark K. Friedberg

**Affiliations:** 1 Department of Cardiology, Hospital for Sick Children, Toronto, Ontario, Canada; 2 Department of Cardiovascular Surgery, Hospital for Sick Children, Toronto, Ontario, Canada; 3 Department of Cardiothoracic and Vascular Surgery & Department of Clinical Medicine, Aarhus University Hospital, Aarhus N, Denmark; University of Buenos Aires, Faculty of Medicine. Cardiovascular Pathophysiology Institute., ARGENTINA

## Abstract

**Background:**

Pulmonary arterial hypertension is usually fatal due to right ventricular failure and is frequently associated with co-existing left ventricular dysfunction. Endothelin-1 is a powerful pro-fibrotic mediator and vasoconstrictor that is elevated in pulmonary arterial hypertension. Endothelin receptor blockers are commonly used as pulmonary vasodilators, however their effect on biventricular injury, remodeling and function, despite elevated isolated right ventricular afterload is unknown.

**Methods:**

Elevated right ventricular afterload was induced by progressive pulmonary artery banding. Seven rabbits underwent pulmonary artery banding without macitentan; 13 received pulmonary artery banding + macitentan; and 5 did not undergo inflation of the pulmonary artery band (sham-operated controls). Results: Right and left ventricular collagen content was increased with pulmonary artery banding compared to sham-operated controls and ameliorated by macitentan. Right ventricular fibrosis signaling (connective tissue growth factor and endothelin-1 protein levels); extra-cellular matrix remodeling (matrix-metalloproteinases 2 and 9), apoptosis and apoptosis-related peptides (caspases 3 and 8) were increased with pulmonary artery banding compared with sham-operated controls and decreased with macitentan.

**Conclusion:**

Isolated right ventricular afterload causes biventricular fibrosis, right ventricular apoptosis and extra cellular matrix remodeling, mediated by up-regulation of endothelin-1 and connective tissue growth factor signaling. These pathological changes are ameliorated by dual endothelin receptor blockade despite persistent elevated right ventricular afterload.

## Introduction

Increased right ventricular (RV) afterload[1] in both acquired and congenital heart disease (CHD) leads to RV injury and dysfunction;[2] and is an important cause of morbidity and mortality in various conditions including severe pulmonary stenosis, tetralogy of Fallot and pulmonary arterial hypertension (PAH). Beyond the effects of increased afterload on the RV itself, the RV and left ventricle (LV) are intimately connected.[3,4] Consequently, RV afterload affects LV function through ventricular-ventricular interactions.[5–7] Hence, the role of LV dysfunction in PAH is increasingly recognized. We previously showed in a rabbit model of isolated increased RV afterload induced by pulmonary arterial banding (PAB) both right and left ventricular myocardial fibrosis and apoptosis.[8] These adverse interactions were associated with up-regulation of several pro-fibrotic signaling molecules, including endothelin-1 (ET-1) and its ET-B-receptor.[8]

ET-1 is a powerful pro-fibrotic mediator and vasoconstrictor[9] that is elevated in PAH.[10,11] Indeed, endothelin receptor blockers (ERB) are currently used as pulmonary vasodilators[12] and the dual A and B ET-receptor antagonist, macitentan, has recently shown to reduce morbidity and mortality in PAH patients.[13] However, in many patients, whether from deficient pulmonary vascular remodeling in PAH, or partial relief of pulmonary or branch pulmonary artery stenosis in CHD, reduction of RV afterload is incomplete and the potential myocardial effects of ERB, independent of their pulmonary vascular effects, is of substantial clinical relevance. Nevertheless, ERB’s direct myocardial effects, independent of their effects secondary to RV afterload reduction, remain undefined. Given the marked biventricular fibrosis in association with up-regulation of ET-1 and its receptors in response to increased RV afterload observed in our prior experiments, myocardial ET-1 blockade, may be an important therapeutic target to improve biventricular remodeling and function, especially when RV afterload cannot be adequately reduced.

Accordingly, the objective of the current study was to investigate the effects of ERB on biventricular remodeling and function in a rabbit model of isolated RV afterload, independent of its effects on the pulmonary vasculature. We hypothesized that macitentan improves biventricular remodeling and function despite persistent RV afterload.

## Materials and Methods

### Ethics statement

Experiments were approved by the Animal Ethics Committee at the Hospital for Sick Children (#19717) and performed in strict accordance with the US National Institutes of Health “Guide for Care and Use of Laboratory Animals” (NIH Publication No. 85–23, revised 1985). All efforts were made to minimize suffering.

### Rabbit model of right ventricular pressure overload

Twenty-five male New Zealand White rabbits with an average preoperative weight of 3 kg had an adjustable band (ABS, Silimed, Brazil)[14] implanted around the main pulmonary artery via left thoracotomy.[8] Animals were pre-anesthetized with ketamine (14.7 mg/kg) and acepromazine (0.3 mg/kg), subcutaneously (sc.), followed by 3% isoflurane via endotracheal tube, maintained at 1.5–2% with continuous monitoring of vital parameters. To reduce pain and suffering following surgery the rabbits received meloxicam (0.3 mg/kg sc.) and buprenorphine (0.05 mg/kg x 3 sc. during the first 24 hours postoperatively. Each rabbit received a single dose penicillin G (3000,000 IU/ml) intramuscular injection (0.2 ml/kg) as prophylaxis against wound infection.

Rabbits were randomized to 3-groups: 1) sham-operated controls (Sham) (n = 5) in which the PAB was left un-inflated, 2) PAB-group (n = 7) with stepwise PAB inflation, and 3) ERB-group (n = 13) with stepwise PAB inflation and the addition of macitentan (Actelion, Allschwil, Switzerland). After a 7-day recovery period following PAB placement, animals in the PAB and PAB+ERB groups underwent stepwise PAB inflation with saline injection to induce gradual RV pressure overload, thereby avoiding death secondary to acute RV failure. PAB inflation was performed in 3 steps at 4-day intervals, aiming for supra-systemic RV pressure after the 3^rd^ inflation. The PAB inflation was monitored by echocardiography for PAB gradient, septal curvature and RV systolic pressure by tricuspid regurgitation Doppler.

Macitentan was started at the time of the 2^nd^ band inflation (~half-systemic RV pressure). It was administered orally once daily (10 mg/kg/day) in a 5% methocel-solution, was initiated after the second PAB inflation (~ half-systemic RV pressure) and continued for an average of 31 days until the terminal experiment. From previous dose-response experiments, 10 mg/kg/day demonstrated the same increased circulating ET-1 concentrations as a higher dose of 30 mg/kg/day.[15]

Three-weeks after the final PAB inflation, rabbits were sedated for the terminal experiment (same method as above) in which RV and LV high-fidelity pressure measurements were performed to assess biventricular function. Thereafter, animals were sacrificed by cardiectomy on anesthesia and tissues harvested for histological and molecular investigations.

### Tissue collection

RV and LV myocardial samples were stored for histological and immunohistochemistry analysis. Myocardial samples from the RV and LV + septum were snap frozen and stored at -80 degrees Celsius for protein analysis by western blotting and real-time polymerase chain reaction (PCR). A second sample was preserved in 10% neutral-buffered formaldehyde and embedded in paraffin for histology. A third sample was snap-frozen in Optimal Cutting Temperature (OCT) compound for cryosection to detect apoptosis.

### Cardiac morphometry

Myocyte size was assessed on 5μm cross-sectional hematoxylin-eosin stained slices. The outer border of transverse sectioned myocytes was drawn and myocyte area calculated (NIH ImageJ). Relative ventricular wall thickness was determined.[16]

Cardiac collagen volume fraction was quantified as the ratio of the sum of total interstitial collagen area to the sum of total collagen and non-collagen areas in the entire visual field of picro Sirius red F3BA (PSR) 5μm stained sections by automated planimetry (Adobe Photoshop CS2, San Jose, CA, USA).

### Blood samples

Circulating ET-1 and macitentan (ACT-064992 and it’s active metabolite ACT-132577) levels were measured from arterial blood drawn from a central line. Plasma was recovered and stored at -80°C (see S1 File for further details).

### Tissue analysis

Western Blots of connective tissue growth factor (CTGF), platelet-derived growth factor (PDGF) and matrix metalloproteinases 2 and 9 (MMP-2 and 9) were performed (see S1 File) as key signaling molecules in cardiac extra-cellular matrix remodeling.[17] Real-time-PCR methods and primers are listed in S1 Table. To confirm MMP activity gelatinolytic zymography was performed, method described in S1 File.

Immunofluorescence staining of ET-1 receptor, myocyte specific α-actinin, and fibrobalst specific vimentin were performed. The methodology is described in S1 File.

### Apoptosis

Terminal deoxynucleotidyl transferase-mediated dUTP nick-end labeling (TUNEL) assay was used to determine RV and LV apoptosis. Apoptosis-related peptides caspases 3 and 8 were evaluated by western blotting (see S1 File for further details).

### Capillary Volume Measurement

Cardiac capillary volume fraction in the RV was determined on CD31 stained section as the ratio of the sum of total capillary area to the sum of total tissue areas in the entire tissue visual field. The images were collected using a microscope (Leica Microsystems), a 20x objective lens was used to collect images, and capillary volume was digitally quantified and expressed as capillary volume percent of total tissue volume in tissue samples.

### Hemodynamics

Systolic and diastolic function including RV and LV maximal rate of ventricular pressure rise (dP/dtmax), end-diastolic pressure (EDP) and the time constant of pressure decay (tau), were measured at the terminal experiment using a 3F high-fidelity micro-manometer catheter (Millar Inc., Houston, TX).

### Echocardiography

The myocardial performance index (MPI) was used as a measure of global RV and LV systolic and diastolic function. M-mode tricuspid annular systolic excursion (TAPSE) and tissue Doppler velocities (TDI) at the tricuspid lateral annulus were used as measures of RV longitudinal function.

### Statistics

Grouped data are presented as mean +/- standard deviation (SD) or as median and range. Groups were compared using one-way ANOVA if data were normally distributed; otherwise non-parametric Kruskal-Wallis testing was used (GraphPad Prism 6.0, San Diego, CA). Post-hoc Holm-Sidak or Dunn’s tests were performed to detect differences between groups. A p-value < 0.05 was considered statistically significant.

## Results

After surgery animals gained weight without signs of heart failure. One rabbit in the ERB-group developed scrotal edema. Water and food were freely available. Intake was normal. The groups had similar age and body weight at initiation and termination of the experimental protocol. One animal in the Sham-group died suddenly in the last week of the protocol.

### Hemodynamics

After the 3^rd^ PAB inflation, RV pressures were elevated as demonstrated by a flattened septum and increased Doppler gradient. At time of the terminal experiment, during anesthesia, catheter measured RV pressures were on average ~2/3 systemic. LV pressures were not significantly different between groups (**Table 1**). RV dP/dtmax trended lower in PAB vs. sham and increased with addition of macitentan but did not reach statistical significance (**Table 1**). LV relaxation (tau) and end-diastolic pressures were not significantly different between groups. RV tau was significantly worsened with PAB (p≤0.05) and decreased in median value in PAB+ERB suggesting improvement by macitentan.

**Table 1:** Hemodynamic and functional characteristics. Conductance and echocardiographic results presented as median (range) as the data is not normally distributed. Pes: end-systolic pressure, Ped: end-diastolic pressure. Sham (n = 3), PAB (n = 5), and PAB+ERB (n = 5). The groups were compared using Kruskal-Wallis multiple comparisons. Post hoc test showed:

LV	Sham (Range)	PAB (Range)	PAB+ERB (Range)
*Conductance*			
dP/dt max *(mmHg/s)*	1536 (1258 to 3307)	1655 (1229 to 2460)	1385 (1152 to 2429)
Tau	24 (12 to 35)	16 (13 to 21)	21 (17 to 30)
Pes *(mmHg)*	45 (44 to 77)	45 (38 to 67)	33 (23 to 76)
Ped *(mmHg)*	13 (7 to 15)	7 (0 to 11)	4 (1 to 25)
*Echocardiography*			
E’ *(cm/s)*	9.2 (7.2 to 11.9)	4.1 (1.6 to5.9)	5.6 (2.7 to 5.3)	
S’ *(cm/s)*	4.8 (4.5 to 7.9)	3.2 (1.1 to 3.7)	4.5 (3.9 to 5.3)	
MPI	0.45 (0.41 to 0.61)	0.43 (0.39 to 0.53)	0.35 (0.27 to 0.39)	
FS *(%)*	32 (21 to 39)	39.5 (26 to 43)	36 (31 to 48)	
**RV**				** **
*Conductance*			
dP/dt max *(mmHg/s)*	571 (484 to 776)	525 (435 to 919)	871 (636 to 2593)
Tau	7 (4 to 20)	28* (27 to 32)	24 (19 to 28)
Pes *(mmHg)*	18 (13 to 18)	24 (21 to 34)	26 (21 to 39)
Ped *(mmHg)*	3 (1 to 6)	10 (4 to 12)	6 (5 to 11)
*Echocardiography*			
E’ *(cm/s)*	12.7 (9.5 to 23.1)	4 (1.8 to 8)	9.8 (2.5 to 13.8)
S’ *(cm/s)*	9.7 (5.5 to 11.2)	3.2** (2 to 3.5)	6 (1.9 to 8.4)
MPI	0.17 (0.12 to 0.47)	0.36 (0.21 to 0.57)	0.28 (0.15 to 0.49)
TAPSE *(cm)*	0.44 (0.31 to 0.53)	0.31 (0.23 to 0.45)	0.38 (0.29 to 0.55)

*RV Tau, the time constant of pressure decay increased with PAB compared to Sham p≤0.05, but did not change with addition of macitentan.

**RV-S’ decreased with PAB compared to Sham p≤0.05, however no significant increase with addition of macitentan.

### Echocardiography

LV-MPI did not change with PAB compared to Sham (**Table 1**), addition of macitentan showed a trending improvement (0.35 (range 0.27 to 0.39) compared to PAB (0.43 (range 0.39 to 0.53), p = ns). LV ejection fraction was preserved in all groups (data not shown). Tricuspid S’ decreased significantly with PAB compared to shams (3.2 cm/s (range 2 to 3.5) vs. 9.7 cm/s (range 5.5 to 11.2 p≤0.05); with a trend towards improvement with macitentan (6 cm/s (range 1.9 to 8.4), p = ns). RV TAPSE trended towards decreasing with PAB compared to Sham (0.31 cm (range 0.23 to 0.45) vs. 0.44 (range 0.31 to 0.53, p = ns) and a trending increase with addition of macitentan (0.38 cm (range 0.29 to 0.55), p = ns). (**Table 1**)

### Plasma endothelin and macitentan

**Fig 1:**
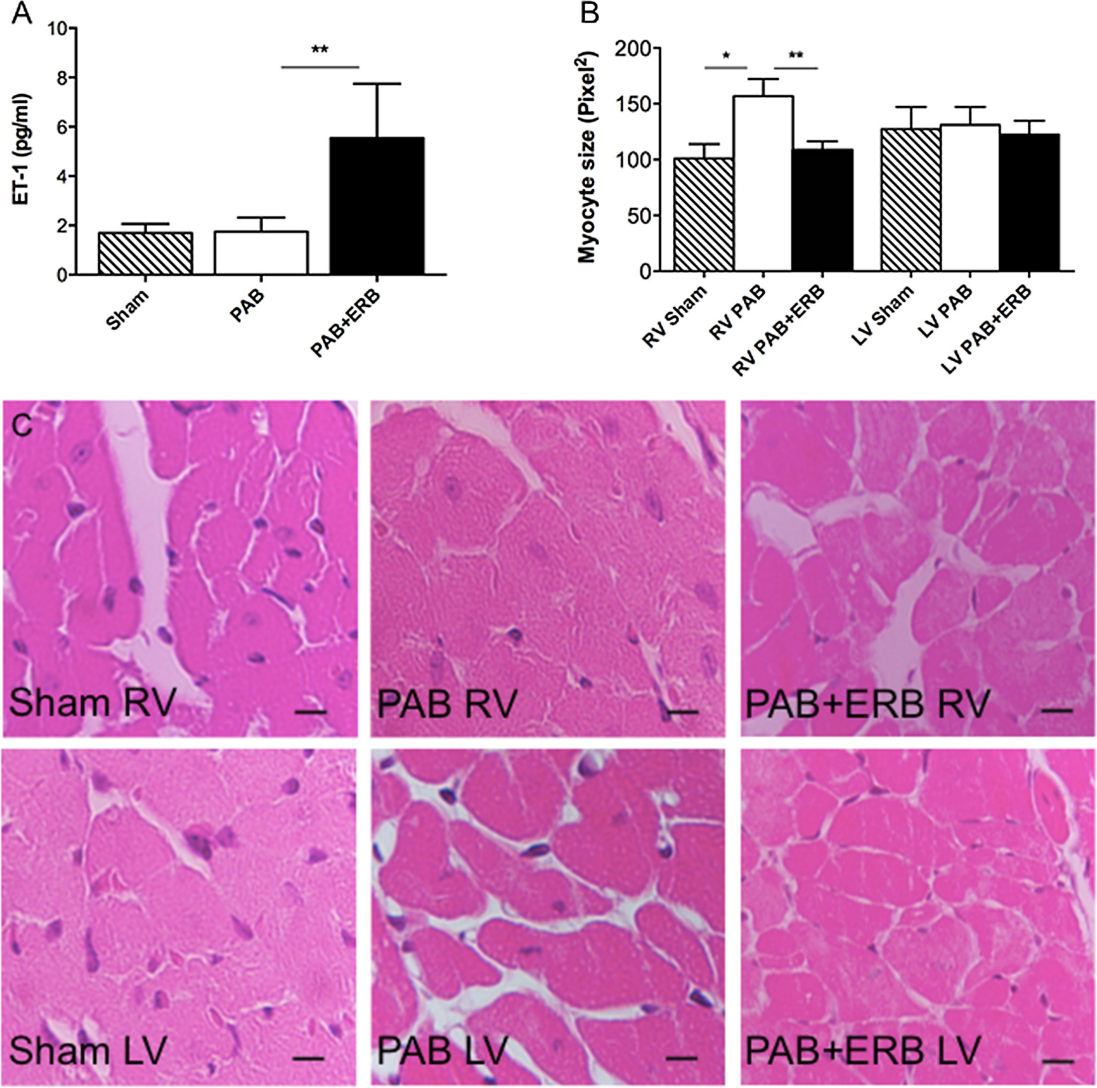
Circulating ET-1, and macitentan levels and ventricular morphology. **A)** Plasma concentrations of endothelin-1 (ET-1) were unchanged in Sham (n = 3) compared to PAB (n = 11), but increased significantly with addition of macitentan (PAB+ERB n = 7) **p≤0.001. **B)** Bar graph showing a significant increase in myocyte size in the PAB group vs. sham for the RV (*p≤0.0001), measured in transverse cut myocytes (n = 4 Sham group, n = 7 for the PAB group, and n = 13 for the PAB+ERB group). Addition of endothelin receptor blocker (PAB+ERB) resulted in a significantly decreased cardiomyocyte size vs. PAB in the RV, ** p≤0.0001. For the LV there were no change in myocyte size. **C)** Hematoxylin-eosin staining, magnification × 40 the black lines is a micron bar indicating 20μm. RV: right ventricle, LV: left ventricle, PAB: pulmonary artery banding, and ERB: endothelin receptor blocker.

Circulating ET-1 levels were similar in the Sham and PAB groups. With addition of ERB, circulating ET-1 levels increased compared to both Sham and PAB, demonstrating biological effect of macitentan in blocking ET receptors (**Fig 1A**).

Plasma-macitentan (ACT-064992) were measured in the PAB+ERB group (n = 11) the median value was 0.54 μg/mL with a range of 0.1 to 2.6 μg/mL. The concentration of the active metabolite of macitentan (ACT-132577) was much larger: 45.9 μg/mL (range 26.8 to 70 μg/mL) showing good absorption and conversion to the active metabolite.

### Ventricular morphology

RV myocyte size was significantly enlarged in PAB animals compared with Sham and significantly lower with macitentan, comparable to Sham values (**Fig 1B and 1C**). There were no significant differences in LV myocyte size between groups.

### Cardiac fibrosis

**Fig 2:**
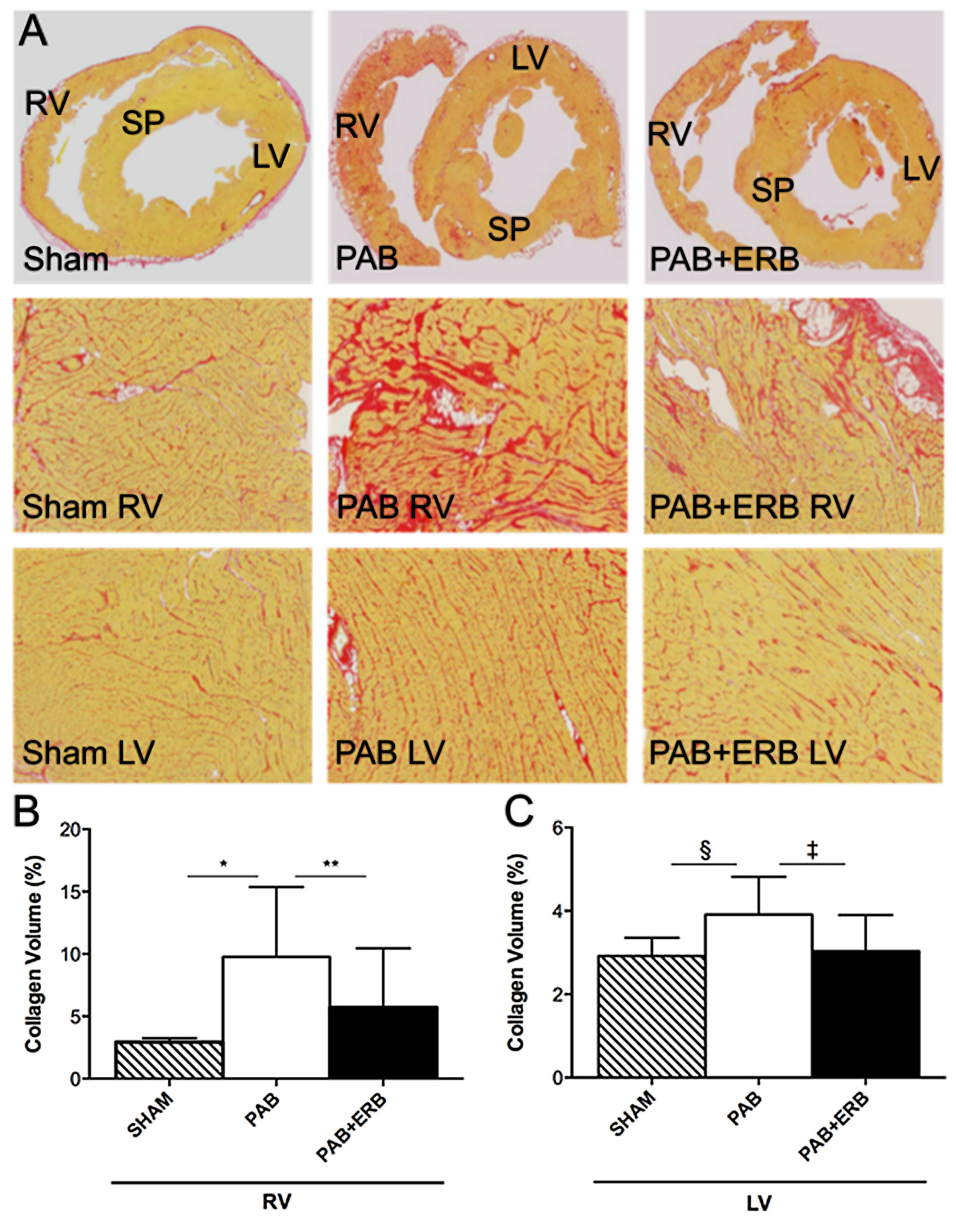
Cardiac Fibrosis. **A)** Picrosirius red staining showed a significant increase of collagen in both the RV & LV in the groups with only pulmonary arterial banding (PAB). Addition of endothelin receptor blocker (PAB+ERB) significantly lowered the amount of collagen in both RV and LV. Magnification ×10. **B)** Quantitative collagen volume fractions in the RV. PAB increased collagen compared with sham (*p≤0.0001), macitentan decreased collagen compared with PAB (**p≤0.0001) **C)** Quantitative collagen volume fractions in the LV. PAB increased collagen compared with sham (§p≤0.0001), macitentan decreased collagen compared with PAB (‡ p≤0.0001). Note the scale difference in the collagen content graphs between the RV and LV. Volume fractions in the various groups were analyzed using Adobe Photoshop (n = 4 for the sham group, n = 7 for the PAB group, and n = 11 for the PAB+ERB group). RV: right ventricle, LV: left ventricle, SP: septum, PAB: pulmonary artery banding, and ERB: endothelin receptor blocker.

RV and LV myocardial collagen content was significantly increased in PAB compared to Sham and significantly lower in ERB animals compared to PAB (**Fig 2**).

### Pro-fibrotic signaling and extra-cellular matrix remodeling

**Fig 3:**
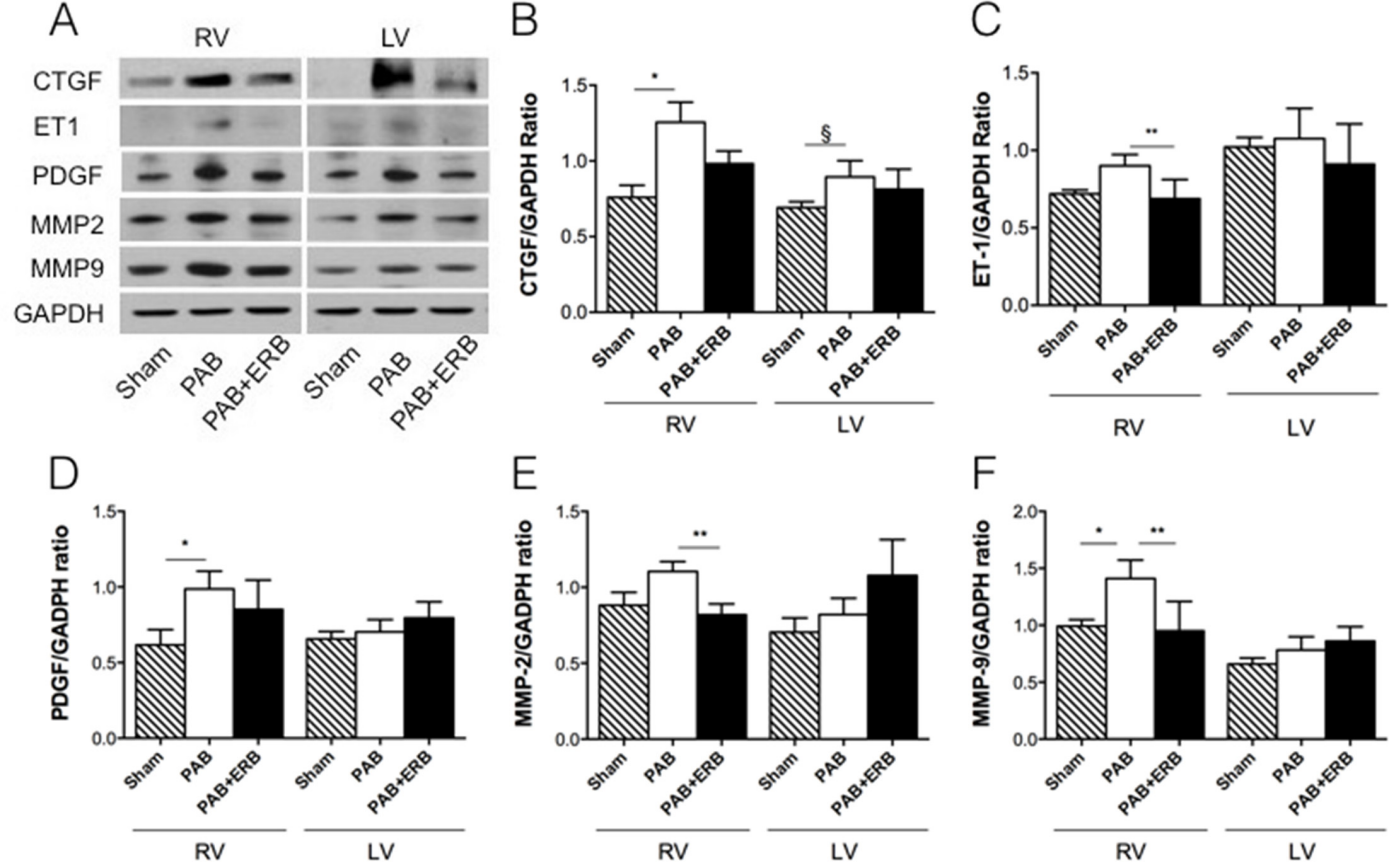
Western blot results of proteins involved in the signaling pathway of fibrosis. Western blot analyses with quantification of Connective Tissue Growth Factor (CTGF), platelet derived growth factor (PDGF), and matrix metalloproteinases 2 and 9 (MMP 2 and 9) proteins level at 6-weeks after PAB. **A)** Western blot examples of the respective proteins. **B)** CTGF increased significantly with PAB compared to sham in the RV (*p≤0.01), in the LV CTGF also increased with PAB compared to sham (§ p<0.05). Addition of macitentan did not significantly decrease CTGF. **C)** the protein level of endothelin-1 (ET-1) decreased significantly with addition of macitentan compared with the PAB group (*p≤0.05). **D)** PDGF increased in the RV with PAB compared with sham (*p≤0.05). **E)** MMP-2 in the RV showed a trending increase with PAB and decreased with addition of macitentan compared with PAB (*p≤0.05) **F)** MMP-9 in the RV increased with PAB compared with sham (*p<0.05), addition of macitentan decreased MMP-9 (**p≤0.05). Glyceraldehyde-3–phosphate dehydrogenase (GAPDH) was used as the internal control. Sham (n = 4), PAB-group (n = 5), and PAB+ERB (n = 5). RV: right ventricle, LV: left ventricle, PAB: pulmonary artery banding, and ERB: endothelin receptor blocker.

**Fig 4:**
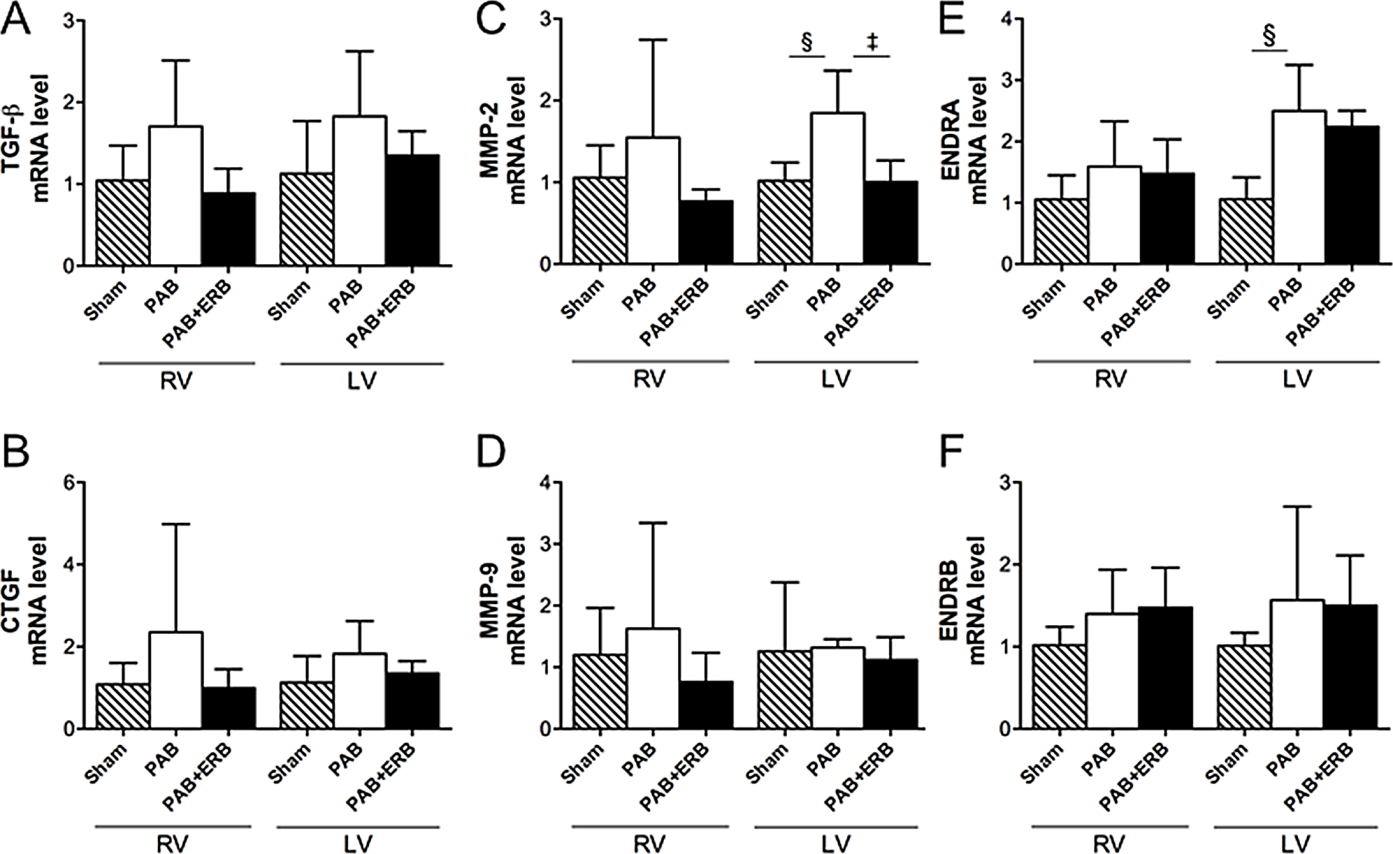
Real-time PCR results of mRNA expression in the myocardium. Real-time PCR data show levels of gene expression in response to endothelin receptor blocker treatment at 6-weeks after PAB. **A & B)** Genes related to profibrotic mediators: transforming growth factor–β1 (TGF-β1) and connective tissue growth factor (CTGF). **C & D)** Matrix metalloproteinase (MMP) -2 and MMP-9 gene expression. In the LV MMP-2 showed significant up-regulation in response to pulmonary arterial banding (sham vs. PAB §p≤0.05) and decrease with addition of macitentan ‡ p≤0.05). **E & F)** Endothelin receptor type A (ENDRA) and endothelin receptor type B (ENDRB); ENDRA was significantly up-regulated in the LV as a response to PAB, §p≤0.01. **G & H)** α- and β-MHC: myocyte heavy chain. Sham (n = 4), PAB-group (n = 5), and PAB+ERB (n = 5). RV: right ventricle, LV: left ventricle, PAB: pulmonary artery banding, and ERB: endothelin receptor blocker.

Western blots and PCR of pro-fibrotic signaling molecules were performed in a subgroup of animals from each group. RV protein levels of connective tissue growth factor (CTGF) were increased in PAB animals compared with Sham, and decreased with addition of ERB vs. PAB **(Fig 3B)**. Transforming growth factor-β1 (TGF-β1) showed the same trend in mRNA expression (TGF-β1 western blots being unavailable in the rabbit) (**Fig 4A**). LV CTGF protein levels **(Fig 3B)** and TGF-β1 mRNA expression **(Fig 4A)** showed the same trend, as in the RV, with increased CTGF and TGF-β1 in the PAB animals compared to Sham and decreased with PAB+ ERB. RV ET-1 protein levels trended to be higher with PAB vs. Sham [p = ns] and were significantly reduced with ERB (p≤0.05 vs. PAB). LV ET-1 protein levels were similar in the 3 groups (**Fig 3C**).

**Fig 5:**
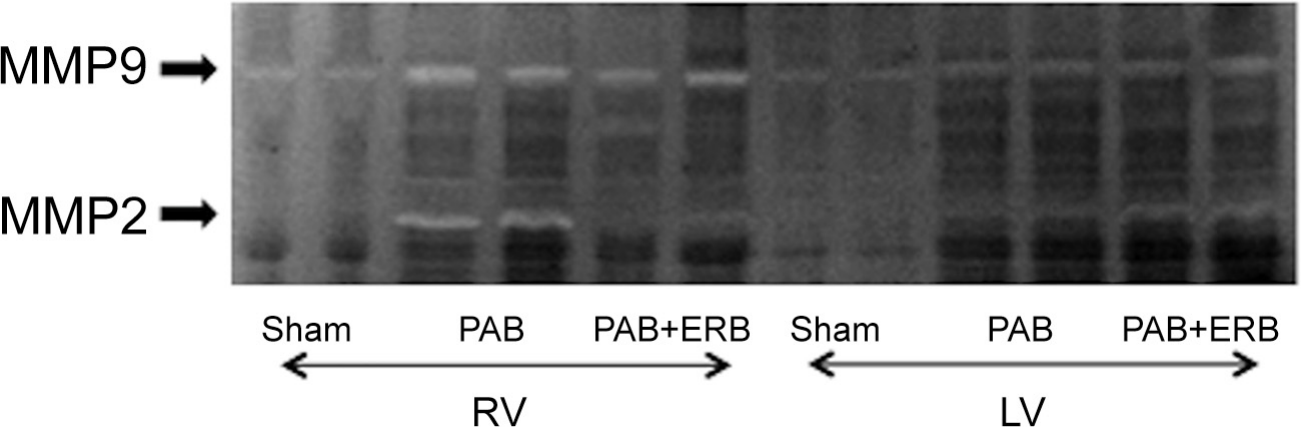
Zymographic image of activated MMP-2 and MMP-9. Representative MMP-9 and MMP2 activity in RV and LV of sham using zymography. Results corresponds with western blot results, showing activated MMP-9 and MMP-2 up regulated in PAB compare with sham; and down regulated after ERB treatment compare with PAB in the RV, but less in the LV.

ET-1 receptor A and B mRNA showed a trending increase with PAB in the RV and a significant increase in the LV, while addition of ERB showed a trending decrease in the LV (**Fig 4E and 4F**). RV PDGF protein levels increased following PAB compared to Sham (**Fig 3D**), and a trending decrease with addition of macitentan (PAB+ERB vs. PAB alone). LV PDGF protein levels were similar between groups. RV matrix metalloproteinases (MMP)-2 and 9 protein levels increased with PAB and decreased with ERB (**Fig 3E and 3F**). LV MMP protein levels were not significantly different between groups, although LV MMP-2 mRNA expression was increased with PAB and lowered with the addition of ERB (**Fig 4C and 4D**). To verify MMP activity gelatinolytic activity assays were performed in both the RV and LV of shams, PAB and PAB+ERB **(Fig 5)**. Results corresponded with western blot results, showing activated MMP-9 and MMP-2 up regulated in PAB compared with sham; and down regulated after ERB treatment compared with PAB in the RV, but less so in the LV.

**Fig 6:**
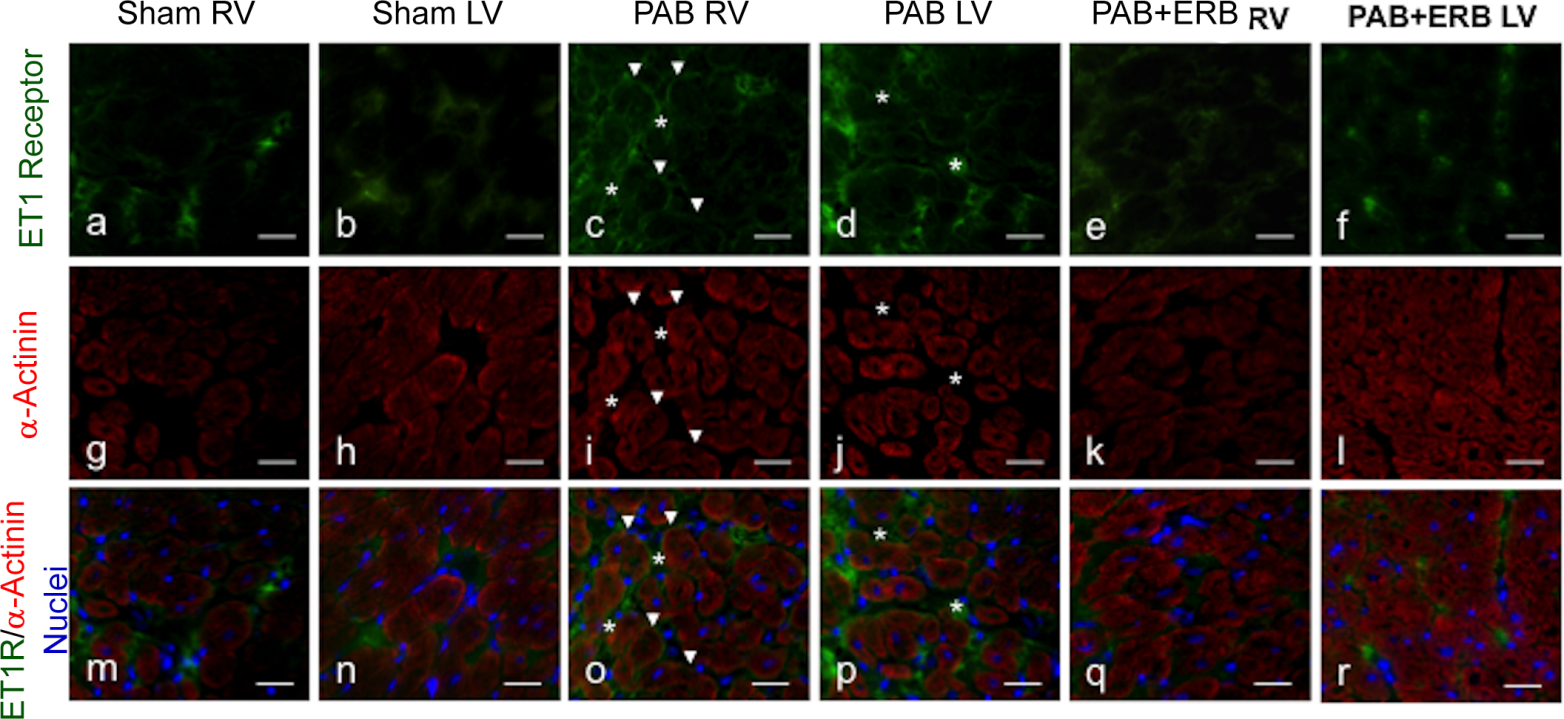
Immunofluoresence staining showing the immunolabeling of ET-1 Receptor of myocytes. Using myocardial samples from sham, PAB, and PAB + ERB treatment from both the RV and LV. Images **a-f** represents ET-1 receptor (in green), images **g-l** (in red) represents α-actinin (myocyte marker). Merged images shown in **m-r**, nucleis are colored blue. The staining shows the expression pattern of ET receptor on cardiomyocytes membrane (arrow), and interstitial area (*). Original magnification, ×400. In sham myocardium, the ET-1 receptor was positively labeled in the membrane of cardiac myocytes and interstitial cells (**a, b**). After PAB, ET-1 receptor strongly labeled myocytes membrane as well as interstitial cells (**c, d**) and decreased after ERB treatment (**e, f**) in both RV and LV.

Immunofluorescence staining shown in **Fig 6** illustrates that ET-1 receptors are present on myocytes and interstitial tissue (**Fig 6A–6F**) verified by co-localization with α-actinin (**Fig 6G–6I**). **Fig 6, m-r** shows merged staining of ET-1 receptors, α-actinin, and nuclei in blue. In sham myocardium, the ET-1 receptor was positively labeled in the membrane of cardiac myocytes and interstitial cells (**a, b**). After PAB, the ET-1 receptor was upregulated in both locations (**c, d**) and decreased after ERB treatment (**e, f**) in both RV and LV.

**Fig 7:**
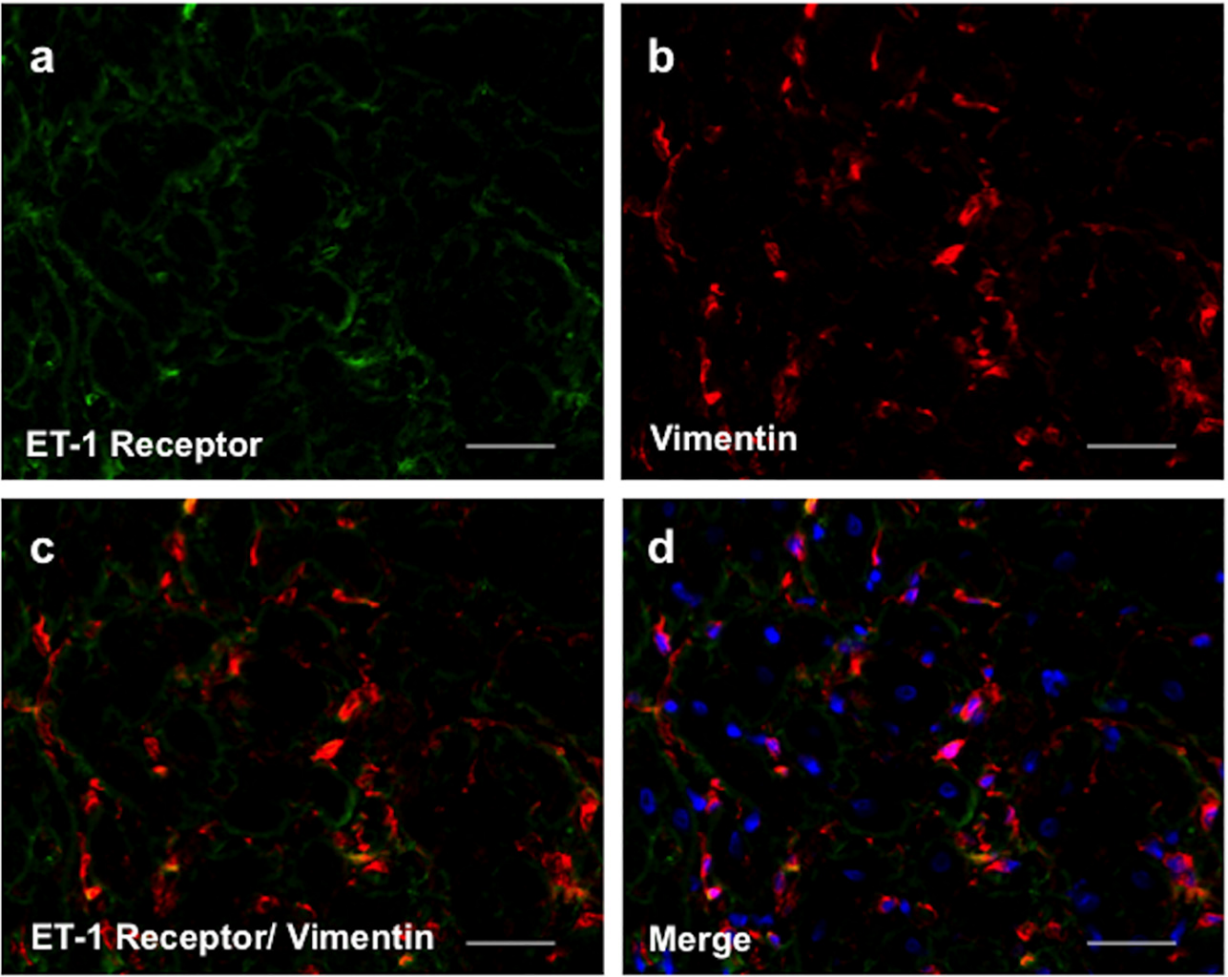
Immunofluoresence staining of ET-1 receptor expressed in cardiac fibroblasts (a-d). (**a**) represent ET-1 receptor (in green) and (**b**) Vimentin, a fibroblast marker (in red) from myocardial samples of the RV from a PAB heart. The orange colour in images **c** and **d** indicates overlapping of ET-1 receptor and Vimentin staining. (**d**) shows the merged image (a and b) including nucleis colored blue. Original magnification, ×400.

Expression of ET-1 receptors on cardiac interstitial fibroblasts is shown in **Fig 7** with immunofluorescence staining of myocardium from the RV of PAB rabbits. Colocalization staining with vimentin demonstrates expression of ET-1 on interstitial fibroblasts.

### Apoptosis

**Fig 8:**
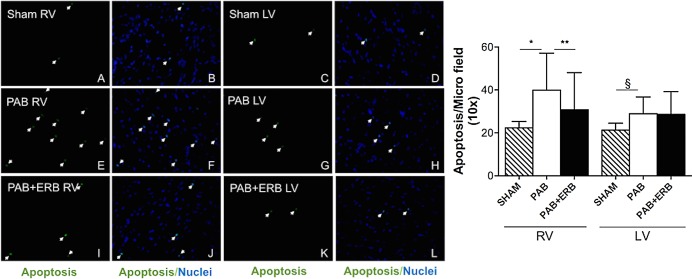
TUNEL results of myocardial apoptosis. **A)** Apoptosis measured by TUNEL assay in sham operated controls (n = 3), in response to pulmonary arterial banding (PAB, n = 3) and endothelin receptor blocker (ERB, n = 4) treatment at 6-weeks after PAB. TUNEL analysis of myocardium from sham RV (A, B), LV(C, D); PAB RV (E, F), LV(G, H); PAB with ERB treatment RV (I, J), LV (K, L) rats heart. Arrows show apoptotic nuclei indicated by green (A, C, E, G, I, K). Blue spots represent nuclear stained by DAPI (4',6-diamidino-2-phenylindole dihydrochloride) and merged images are shown in B, D, F, H, J and L (magnification ×400). **B)** Bar graph with quantification of apoptosis in the right and left ventricles. In the right ventricle apoptosis increased with PAB compared to sham (*p≤0.0001) and diminished with macitentan (compared with PAB **p≤0.05). For the LV, apoptosis increased with PAB compared with sham (*p≤0.01), but there were no significant changes with macitentan. RV: right ventricle, LV: left ventricle, PAB: pulmonary artery banding, and ERB: endothelin receptor blocker.

**Fig 9:**
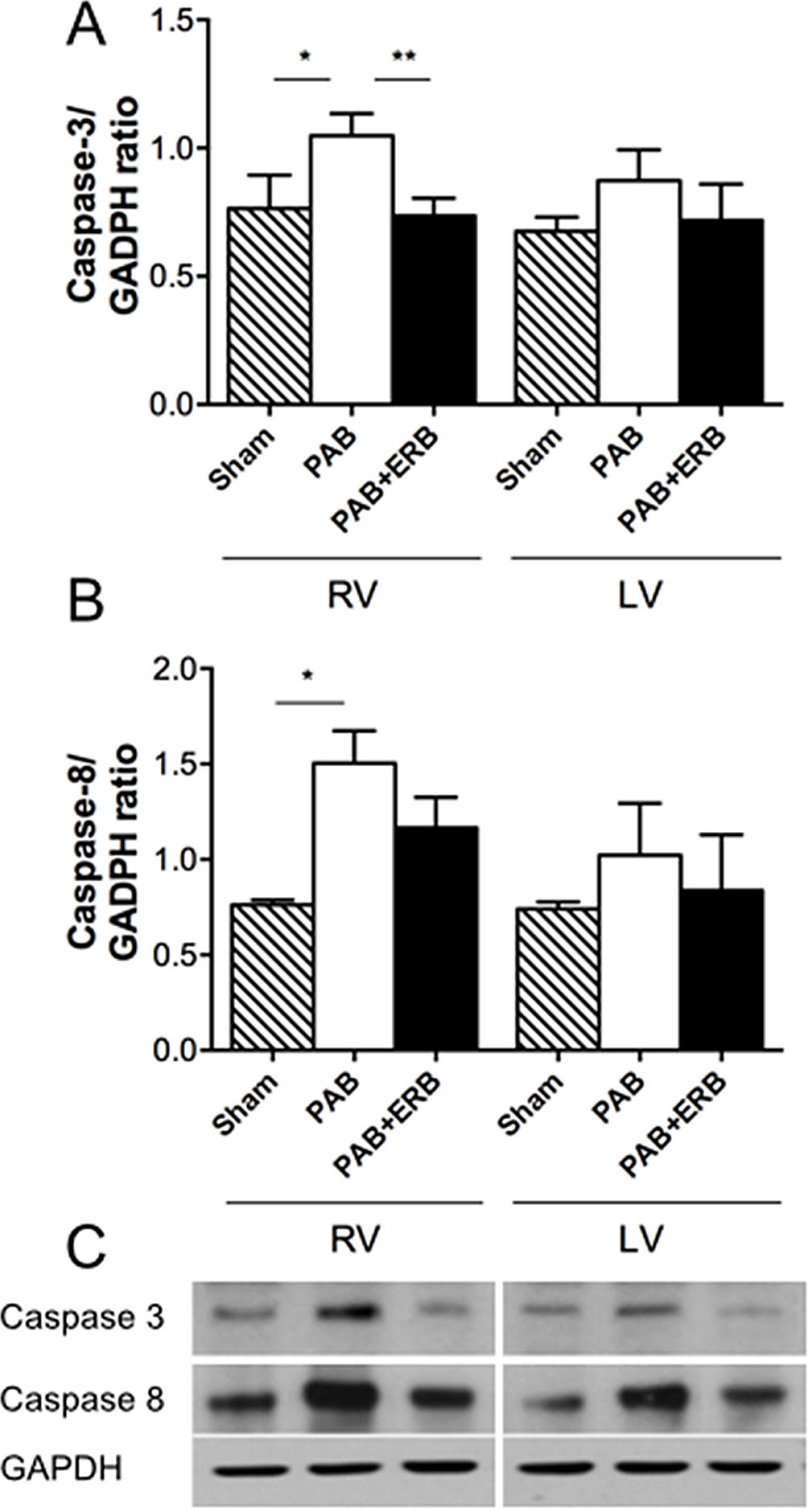
Western blot results of Caspase 3 and 8, peptides involved in apoptosis. Illustrated in the graphs are the quantification of Caspase 3 and 8. **A)** Shows caspase 3 increase with PAB compared with sham (*p≤0.05) and diminish with macitentan compared with PAB (**p≤0.05). **B)** Shows caspase 8 increase with PAB compared to sham (*p≤0.01) however, macitentan did not significantly diminish Caspase 8. **C**) Western blot examples are shown below the graphs of the respective proteins. Glyceraldehyde-3–phosphate dehydrogenase (GAPDH) was used as the internal control. Sham (n = 4), PAB-group (n = 5), and PAB+ERB (n = 5). RV: right ventricle, LV: left ventricle, PAB: pulmonary artery banding, and ERB: endothelin receptor blocker.

RV apoptosis assessed by TUNEL assay was significantly increased in PAB compared to Sham; addition of macitentan significantly reduced RV apoptosis. LV apoptosis was increased in the PAB group vs. Sham but not changed with macitentan (**Fig 8B**). Caspase-3 and -8 protein levels corresponded to TUNEL results: RV Caspase-3 and 8 protein levels significantly increased following PAB, and Caspase 3 was significantly decreased following the addition of macitentan, whereas Caspase 8 only showed a trending decrease. LV caspase-3 and -8 protein levels were not significantly different between groups (**Fig 9**).

### Cardiac vessel volume

**Fig 10:**
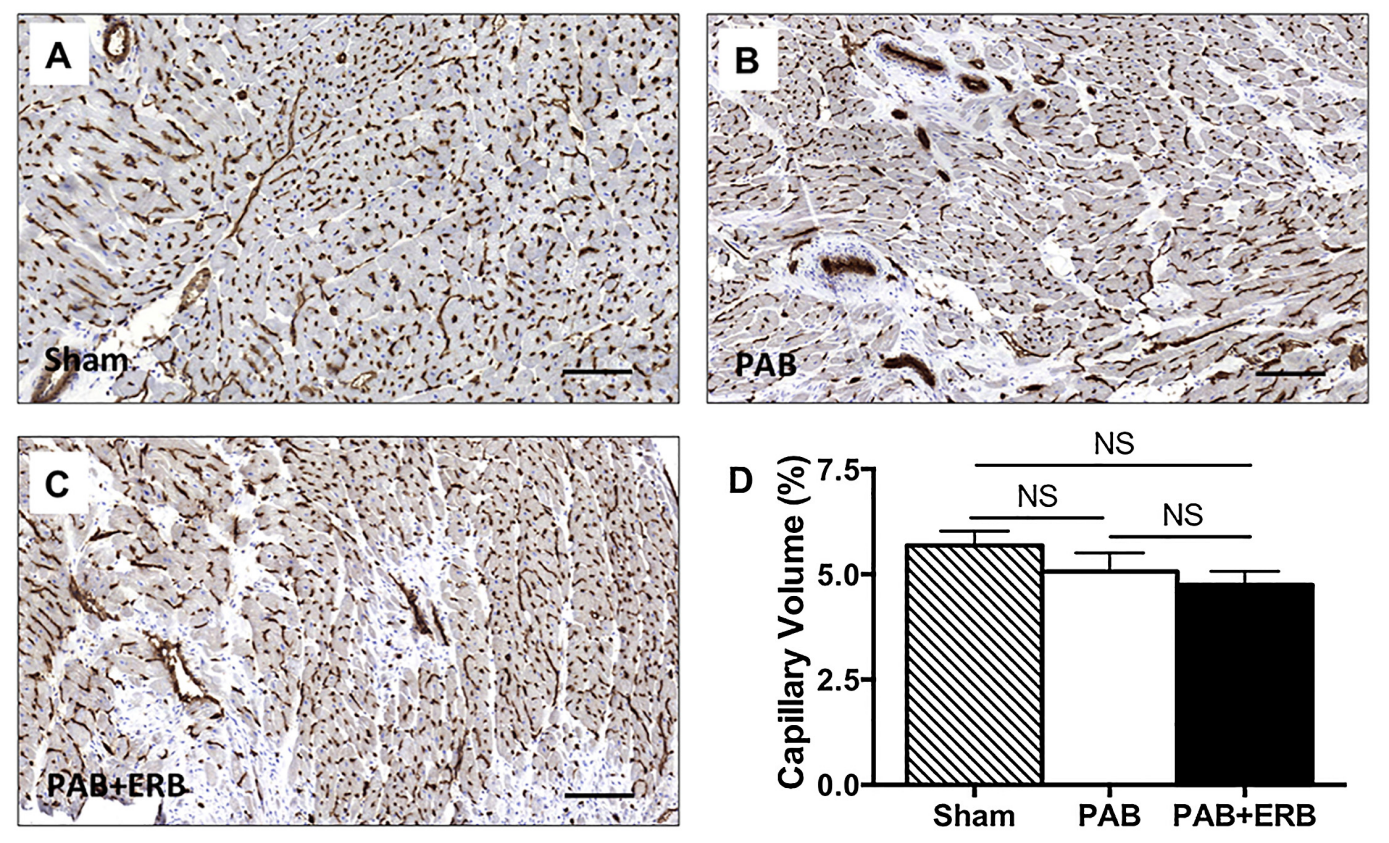
Immunostaining of CD31 represented cardiac vessel volume in sham (A), PAB (B), and PAB+ERB (C) The black lines indicate 100μm. D) Quantification of capillary volume (expressed as percentage of tissue volume) shows the PAB group compared to sham, and the PAB+ERB group compared to sham or PAB, did not change. Sham (n = 4), PAB-group (n = 4), and PAB+ERB (n = 4).

Quantification of capillary volume did not reveal changes with PAB compared to sham or with PAB+ERB compared to sham or PAB. (**Fig 10**)

## Discussion

ERBs are used as pulmonary vasodilators in PAH[12], but their direct effects on biventricular myocardial remodeling and injury in increased RV pressure-load, independent of pulmonary vascular resistance, is poorly characterized. We previously demonstrated that increased RV pressure-load induces biventricular myocardial fibrosis and apoptosis in association with ET-1, TGF-β1 and CTGF signaling.[8,18] In the current study, we show that these effects, especially fibrosis, can be ameliorated by the dual endothelin receptor blocker macitentan, in association with improved biventricular function and independent of its effects on pulmonary vascular resistance. These results are therefore pertinent to a wide array of clinical conditions where RV afterload is persistently increased including PAH and various CHD.

### RV overload model

Our rabbit PAB model offers several advantages, as rodent models that use hypoxemia and/ or pharmacological agents to increase pulmonary vascular resistance, do not differentiate direct myocardial effects of ET-1 blockade from those secondary to its profound effects on pulmonary vascular resistance. In our model, echocardiography during serial inflation of the PAB demonstrated significantly increased RV systolic pressure through a flat interventricular septum and high PAB gradient. In response to the increased afterload, the hemodynamic parameters suggest a partial or early adaptive RV response rather than frank RV failure. This is consistent with previous models of isolated PAB and very pertinent to the clinical development of increased RV afterload in various conditions including the earlier stages of PAH, pulmonary or conduit stenosis, tetralogy of Fallot and others.[19] Nonetheless, while the model did not produce the florid maladaptive response of end-stage RV failure, important adverse effects of PAB were readily apparent in increased RV myocyte size, fibrosis and apoptosis and impaired RV-relaxation, and decreased tissue Doppler velocities. These findings are important as many patients with increased RV afterload, whether from PAH, pulmonary stenosis or various CHD, do not manifest end-stage RV failure until late in the clinical course, but progressive myocardial injury may be an important therapeutic target before the development of irreversible RV failure. Our findings of extensive tissue injury also suggest that RV pressures may have been significantly higher for the duration of the experiment than the 2/3 systemic RV pressures measured at the terminal experiment under profound anesthesia.

### Myocardial fibrosis and apoptosis

Cardiac fibrosis is emerging as an important pathophysiological entity in various settings.[17,20,21] However, relatively little is known about the biventricular biochemical and histological consequences of increased RV afterload and the development of biventricular fibrosis under these circumstances. Adverse effects of increased RV afterload, especially fibrosis, were improved with macitentan, despite similar RV pressures, and independent of pulmonary vascular resistance. We previously showed that isolated RV pressure-load leads to a largely similar pattern of pro-fibrotic molecular signaling, fibrosis and apoptosis in the left and right ventricles.[18] These were associated with increased pro-fibrotic signaling, including increased myocardial ET-1 expression. Hence, ET-1 was a logical target to reduce biventricular myocardial injury, especially as ERBs are clinically used as pulmonary vasodilators in PAH. We specifically used macitentan in this study because of its superior tissue penetration, longer half-life, and previous reports of reduced RV hypertrophy in a monocrotaline PAH rat model, although that effect may have been secondary to reduced pulmonary vascular resistance.[15] Macitentan blocks endothelin A and B receptors, thereby increasing circulating endothelin blood levels. The increase in plasma endothelin concentration has previously been used as an indicator of endothelin receptor blockade. [15,22]

Our data provides preliminary insights into the molecular mechanisms of biventricular fibrosis in response to increased RV afterload. ET-1 levels are consistently elevated in experimental models of cardiac fibrosis[20] and ET-1 interacts with other signaling molecules, including TGF-β1 and CTGF to induce fibrosis and extra-cellular matrix remodeling.[23] Our previous studies and others, showed that biventricular fibrosis in response to isolated RV afterload is mediated through biochemical crosstalk involving the TGF-β1−ET-1−CTGF axis; [8,18,21] Our current results, suggest that ET-1 is a key mediator of biventricular fibrosis in response to isolated RV afterload and that its blockade reduces biventricular myocardial fibrosis. Consistent with current concepts, the similar circulating ET-1 levels between Sham and PAB animals may suggest a local or paracrine effect, although the possibility of circulating ET-1 driving biventricular fibrosis needs further study.[17]

From our tissue results of Western and PCR analyses we can only describe a trend in the signaling pathway as the power of included animals limit the statistical outcome. We believe that following ET-1 receptor blockade, circulating ET-1 levels significantly increase while CTGF protein levels and TGF-β1 mRNA expression, thought to act upstream of ET-1, shows a decreasing trend. These molecular interactions however are likely important in that ET-1 interacts with CTGF, TGF-β1, PDGF and other profibrotic molecular signaling.[17] Therefore, inhibiting ET-1 exerts a wider effect to down regulate the broader profibrotic axis as suggested by our observed results of decreased TGF-β1 and CTGF signaling. In addition, macitentan may affect post-transcriptional modification of ET-1 signaling, but this was not investigated here and requires further study.

In our study, these molecular changes were associated with an array of histological effects. Beyond collagen deposition per se, our results suggest that increased RV afterload induces a broader cascade of myocardial injury involving ECM remodeling and apoptosis. RV MMP-2 and -9 protein levels increased with PAB and normalized with ERB, despite persistent RV afterload. This suggests that ET-1 is an important promoter of ECM remodeling in response to increased RV afterload. Likewise, apoptosis, which is a central mechanism of myocardial injury, has received little attention in increased RV afterload.[24] While our results do not demonstrate the downstream signaling pathways mediating the effects of ERB on apoptosis, our results are congruent with those of recent studies showing that knockout of the ET-1 A receptor mitigates toxin induced cardiac apoptosis.[25] Protein expression studies derived from pulmonary arterial smooth muscle cells from PAH patients reveal up-regulation of pathways linked to apoptosis including eIF2/mTOR/p70S6K, RhoA/actin cytoskeleton/integrin and protein unbiquitination proteins; which were further up-regulated with ET-1.[26] Further studies are needed to elucidate the mechanism of ERBs effects on myocardial apoptosis.

Angiogenesis in the myocardium assessed by capillary volume was not affected by PAB or addition of macitentan. Addressing angiogenesis in further detail in future studies is very pertinent.

Improved RV myocardial injury indicated a trending improvement in RV function, whereas the functional benefits of improved LV myocardial injury were absent. In PAB animals, LV ejection fraction was preserved in all groups despite the significant increase in LV fibrosis. In this situation it is difficult to demonstrate improvement with treatment and more severe RV afterload or a longer disease duration may be required. Similar results were shown by Kitahori et al.[7] in a juvenile rabbit PAB model where septal and LV apoptosis were associated with LV diastolic dysfunction but preserved ejection fraction.

### Clinical implications

ERBs improve hemodynamics, functional class, morbidity and mortality in PAH patients. These effects are attributed mainly to improved pulmonary vascular resistance (PVR).[12,13,27] However, as previously discussed, while some studies suggest that ERB reduce RV hypertrophy and fibrosis in rats with elevated RV afterload,[15,17] their direct myocardial effects, independent of their pulmonary vasodilator effects, have not been well studied. Given the importance of ventricular function and fibrosis to outcomes in PAH, tetralogy of Fallot and other conditions of increased RV afterload, the beneficial biventricular myocardial effects of macitentan, observed in our study, independent of its impact on pulmonary vascular resistance may be an important consideration in future treatment algorithms of conditions characterized by persistently elevated RV afterload. The functional effects of our findings are clearly preliminary and require further investigation. However, the trend towards improved RV dP/dtmax may suggest improved RV contractility and at the least no worsening of RV contractility with macitentan, as suggested by previous studies during bosentan treatment.[28] Furthermore, the trend towards reduced biventricular EDP with macitentan, in association with reduced fibrosis is clinically relevant, as increased fibrosis and filling pressures increase risk for ventricular dysfunction, exercise intolerance, clinical arrhythmias, neuro-hormonal activation, and mortality in PAH and CHD.[29]

### Study limitations

Despite repeated attempts with several different antibodies we were unable to measure TGF-β1 protein levels in the rabbit. TGF-β1 mRNA levels showed a trending decrease following macitentan and were in keeping with decreased protein levels of CTGF, known to act downstream of TGF-β1. We therefore believe that TGF-β1 mRNA expression levels are indicative of protein activity.

ERB was started during incremental increase in RV pressure. Therefore, observed effects may reflect prevention, rather than reversal of existing fibrosis and ECM remodeling. This requires further study, but may indicate that earlier therapy may be beneficial. As per convention, LV analysis included the interventricular septum. Therefore, we cannot exclude that LV effects may be due to septal effects.

In conclusion, isolated right ventricular afterload causes biventricular fibrosis, right ventricular apoptosis and extra cellular matrix remodeling, mediated by up-regulation of endothelin-1, connective tissue growth factor signaling, and matrix metalloproteinases (2 and 9). These pathological changes are ameliorated by dual endothelin receptor blockade despite persistent elevated right ventricular afterload.

## Supporting Information

S1 File Method. (DOCX)

S1 Table Table A. (DOCX)
