# Supplementary material for: Dual Endothelin Receptor Blockade Abrogates Right Ventricular Remodeling and Biventricular Fibrosis in Isolated Elevated Right Ventricular Afterload
Source: PLoS One. 2016 Jan 14;11(1):e0146767. doi: 10.1371/journal.pone.0146767 (PMC4713098; doi:10.1371/journal.pone.0146767)
Supplement: S1 File — (DOCX) [file pone.0146767.s001.docx]

**Supplement information**

Methods:

Blood samples

Arterial blood samples were drawn from a central line into vacutainers containing 1.8 mg/ml EDTA (ethylenediaminetetraacetic acid). Plasma was recovered and stored at -80°C. Circulating endothelin-1 (ET-1) levels were determined by direct chemiluminescent ELISA (enzyme-linked immunosorbent assay) using ET-1 QuantiGlo (QET00B R&D systems, Minneapolis, MN, USA). Quantification of macitentan and ACT-132577 was determined by liquid chromatography coupled to mass spectrometry (API4000, AB SCIEX, Concord, Ontario, Canada).

Western Blot Analysis

Cardiac tissues samples were homogenized with lysis buffer and diluted 1:1 with 2×SDS sample buffer (Invitrogen Novex, Carlsbad, CA, USA). An equal amount of protein (30μg) was loaded onto each lane of an 8% to 16% Tris-Glycin gel (Helixx). Proteins were separated by electrophoresis and transferred to a nitrocellulose membrane using an electro-blotting apparatus (Invitrogen, Carlsbad, CA, USA). Membranes were incubated with 5% Bovine Serum Albumin (BSA) for 1 hour to decrease nonspecific binding. Samples were then incubated with the following primary antibodies overnight at 4°C: Connective Tissue Growth Factor (CTGF) (Abcam, Cambridge, UK), platelet-derived growth factor (PDGF) (Santa Cruz Biotechnology, Inc., Dallas, TX, USA) and matrix metalloproteinases 2 and 9 (MMP-2 and 9) (Abcam, Cambridge, UK). Samples were washed and incubated with peroxidase – conjugated secondary antibody, and detected using the Amersham ECL (GE life sciences, Buckinghamshire, UK) detection kit. Glyceraldehyde 3-phosphate dehydrogenase (GADPH) was used as the internal standard.

Real-time RT-PCR analysis

Cardiac pro-fibrotic gene expression levels were measured by the StepOnePlus^TM^ real-time PCR system (Life Technologies, Carlsbad, CA, USA) using Power SYBR® Green PCR Master Mix (Life Technologies, Carlsbad, CA, USA). Real-time PCR was performed as follows: 10 min at 95°C, 40 cycles of 95°C for 15 sec., followed by 60°C for 1 min. GAPDH was used as internal standard. Primer sequences are listed in **Table 1**. Cycle threshold values (C_t_) were provided by the software, and the 2^-ΔΔCt^ method of relative quantification utilized to compute relative expression levels.

Zymography

Protein was extracted with lysis buffer following homogenization. Samples (30 μg) were loaded onto each lane of 10% zymogram gelatin minigels (Novex, San Diego, CA). Gels were run at 35 mA for 2.5 h and then incubated in 2.5% Triton-X 100 for 2× 1 h at room temperature, washed, and further incubated for 16 h in 50 mmol Tris-HCl buffer, pH 7.5, containing 200 mmol NaCl and 10 mmol CaCl2 at 37°C. Gels were stained for 90 min in Coomassie blue and destained in 30% methanol/10% acetic acid for 60 min. White bands on a blue background indicated zones of digestion corresponding to the presence of different matrix metallopeptidases (MMPs) identified on the basis of their molecular weight. Bands were scanned using a densitometer (GS-700; Bio-Rad, Hercules, CA). MMP levels were quantified using the Multianalyst software (Bio-Rad).

Immunofluorescence

Cryostat sections (5-μm) were prepared, air-dried, and fixed in 4% paraformaldehyde/PBS for 15-minutes. Sections were incubated with 0.3% hydrogen peroxide and 10% BSA for 15-minutes. Sections were then incubated with antibody against ET-1A receptor (abcam Inc) and vimentin (Cell signaling), and A-actinin (Sigma Aldrich) at 4°C overnight. Sections were then incubated with an appropriate fluorescein-conjugated secondary antibody for 60-minutes at room temperature. Negative controls were performed for all immunological staining by omission of the primary antibody. Nuclei were counterstained with DAPI (4',6-diamidino-2-phenylindole dihydrochloride).

Determination of apoptosis

The terminal deoxynucleotidyl transferase-mediated dUTP nick-end labeling (TUNEL) assay was used to monitor apoptotic cells in cryostat sections according to manufacturer recommendations (Boehringer Mannheim, USA). Fluorescein-conjugated dUTP incorporated in nucleotide polymers was detected and quantified by fluorescence microscopy. TUNEL-positive nuclei were distinguished from the TUNEL-negative nuclei by counterstaining with Hoechst 33258 (Sigma, Canada), which were then photographed and counted. The count was done blindly. The percentage of nuclei labeled by TUNEL per unit of cells stained with Hoechst nuclear dye reflected the apoptotic index.
