# Supplementary material for: Dual Endothelin Receptor Blockade Abrogates Right Ventricular Remodeling and Biventricular Fibrosis in Isolated Elevated Right Ventricular Afterload
Source: PLoS One. 2016 Jan 14;11(1):e0146767. doi: 10.1371/journal.pone.0146767 (PMC4713098; doi:10.1371/journal.pone.0146767)
Supplement: S1 Table — (DOCX) [file pone.0146767.s002.docx]

Table A. Polymerase chain reaction, oligonucleotid primers.

| mRNA | Primer sequence |
| --- | --- |
| TGF-β (forward) | 5’-AGGGCTACCACGCCAACTT-3’ |
| (reverse) | 5’-CCGGGTTGTGCTGGTTGTAC-3’ |
| CTGF (forward) | 5’-CCCTGCGTCTTCGGTGGC-3’ |
| (reverse) | 5’-AGGCAGTTGGCTCGCATCAT-3’ |
| MMP-2 (forward) | 5’-AGGACTACGACCGCGACAAG-3’ |
| (reverse) | 5’-TGTTGCCCAGGAAGGTGAAG -3’ |
| MMP-9 (forward) | 5’-CTTCCAACTTTGACAGCGACA-3’ |
| (reverse) | 5’-GGAGTGATCCAAGCCCAGTG-3’ |
| Alpha MHC (forward) | 5’-AGAAGCACGCAACCGAGAA-3’ |
| (reverse) | 5’-TCCTCAGCCTGAAGGTCATCTAG-3’ |
| Beta MHC (forward) | 5’-GACGGTGGTGGCCCTGTAC-3’ |
| (reverse) | 5’-CCTTGCCTTTGCCCTTCTC-3’ |
| ENDRA (forward) | 5’-GCTTCTTGCTGCTCATGGATTAC-3’ |
| (reverse) | 5’-CCGAGGTCATCAGGCTCTTG-3’ |
| ENDRB (forward) | 5’-CTGGCCATTTGGAGCTGAGA -3’ |
| (reverse) | 5’-TTTGGAACCCCAATTCCTTTAA -3’ |
| GADPH (forward) | 5’-AGGCCGTGGGCAAGGT-3’ |
| (reverse) | 5’-CCTCGGATG CCTGCTTCA-3’ |
